# Adenosine-5'-triphosphate (ATP) supplementation improves low peak muscle torque and torque fatigue during repeated high intensity exercise sets

**DOI:** 10.1186/1550-2783-9-48

**Published:** 2012-10-09

**Authors:** John A Rathmacher, John C Fuller, Shawn M Baier, Naji N Abumrad, Hector F Angus, Rick L Sharp

**Affiliations:** 1 Department of Animal Science, Iowa State University, Ames, IA, 50010, USA; 2 Metabolic Technologies Inc, Iowa State University Research Park, Ames, IA, 50010, USA; 3 Department of Surgery, Vanderbilt University School of Medicine, Nashville, TN, 37232, USA; 4 Department of Kinesiology, Iowa State University, Ames, IA, 50010, USA

**Keywords:** ATP, Adenosine-5’-triphosphate, Muscle strength, Muscle fatigue

## Abstract

**Background:**

Intracellular concentrations of adenosine-5’-triphosphate (ATP) are many times greater than extracellular concentrations (1–10 m*M* versus 10–100 n*M*, respectively) and cellular release of ATP is tightly controlled. Transient rises in extracellular ATP and its metabolite adenosine have important signaling roles; and acting through purinergic receptors, can increase blood flow and oxygenation of tissues; and act as neurotransmitters. Increased blood flow not only increases substrate availability but may also aid in recovery through removal of metabolic waste products allowing muscles to accomplish more work with less fatigue. The objective of the present study was to determine if supplemental ATP would improve muscle torque, power, work, or fatigue during repeated bouts of high intensity resistance exercise.

**Methods:**

Sixteen participants (8 male and 8 female; ages: 21–34 years) were enrolled in a double-blinded, placebo-controlled study using a crossover design. The participants received either supplemental ATP (400 mg/d divided into 2 daily doses) or placebo for 15 d. After an overnight fast, participants underwent strength and fatigue testing, consisting of 3 sets of 50 maximal knee extensions performed on a Biodex® leg dynamometer.

**Results:**

No differences were detected in high peak torque, power, or total work with ATP supplementation; however, low peak torque in set 2 was significantly improved (p < 0.01). Additionally, in set 3, a trend was detected for less torque fatigue with ATP supplementation (p < 0.10).

**Conclusions:**

Supplementation with 400 mg ATP/d for 15 days tended to reduce muscle fatigue and improved a participant’s ability to maintain a higher force output at the end of an exhaustive exercise bout.

## Background

The intracellular role of ATP as the energy source for tissues has long been recognized [1]. However, the extracellular metabolic functions of ATP have only recently been investigated, and primary to this function is the role of ATP in signal transduction through purinergic receptors found in most cell types [2]. Extracellular functions of ATP include vasodilation [3] and reduced pain perception [4]. Additionally, ATP is often referred to as a cotransmitter that affects local tissue changes in neurotransmission and neuromodulation by acting upon both peripheral and central nervous systems [5,6].

Whereas intracellular concentrations of ATP are relatively high (1-10 *mM*), extracellular concentrations are tightly regulated at very low levels (10-100 *nM*) [7,8]. When ATP is infused into the arterial blood flow of muscle, the half-life has been shown to be <1 second [9] as ATP is rapidly degraded to adenosine by several surface-expressed and soluble enzymes of the ectonucleoside families [10]. ATP in blood is primarily carried by erythrocytes [8]. Therefore, measurement of circulating free plasma ATP derived from oral supplementation may not be possible as exogenous free ATP or its metabolite adenosine are quickly taken up by blood components. In rats chronic oral administration of ATP at 5 mg/kg/day increased portal vein ATP concentration and nucleoside uptake by erythrocytes which resulted in an increase in ATP synthesis in the erythrocytes [11]. Therefore, the possibility exists for oral ATP to elicit metabolic effects despite an apparent lack of increased systemic free ATP concentrations.

Adenosine, resulting from the degradation of ATP, may also act as a signaling agent through purinergic receptors [12] which are ubiquitously present in many cell types including smooth muscle, endothelial, and neural [2]. Adenosine may further be degraded by adenosine deaminase [10]. The labile state of ATP and its metabolite adenosine cause hyperpolarization and vasodilation in the arteriolar tree resulting in increased blood flow through the tissue, which aids in the removal of waste products such as lactate [13]. For example, signaling by both ATP and adenosine plays an important role in increasing blood flow by causing dilation of the microvasculature when released from erythrocytes passing through the capillaries [13,14]. Adenosine’s effect is also mediated by increased nitric oxide and prostacyclin levels in microvascular endothelial cells [15]. Diverting some of the blood flow also assures the most efficient flow of cardiac output through the exercising muscle. In a similar manner, the release of endogenous ATP from cardiomyocytes occurs in response to ischemia [16], thus resulting in increased blood flow and increased oxygen and glucose delivery to the active muscle tissue. These observations lead to the hypothesis that dietary supplementation with ATP (and/or adenosine) should be beneficial to exercising muscle tissue. However, it should be noted that it is unlikely that ATP is absorbed intact in humans [17,18] and the effect of oral ATP on muscle performance is likely due to the previously described purinergic signaling [2] or through ATP metabolites such as adenosine [12,19].

Supporting this hypothesis of purinergic signaling, Calbet et al. demonstrated that infusion of ATP at near-maximal exercise resulted in increased blood flow to less-active and non-muscle tissues [20]. Improving blood flow through less active muscle tissues could remove waste products such as lactate. Additionally, Jordan et al. demonstrated that orally ingested ATP may be metabolically available to tissues and may influence adenine nucleotide metabolism during exercise [21]. The study showed that oral supplementation with ATP (225 mg) for 14 days resulted in increased within group set-one repetitions and increased total lifting volume on the bench press apparatus; however, no effect was observed at the lower dosage of 150 mg ATP per day. The current study was designed to test the hypothesis that supplemental ATP would improve performance of repeated high intensity exercise as measured by muscle torque, power, work and fatigue.

## Methods

**Figure 1:**
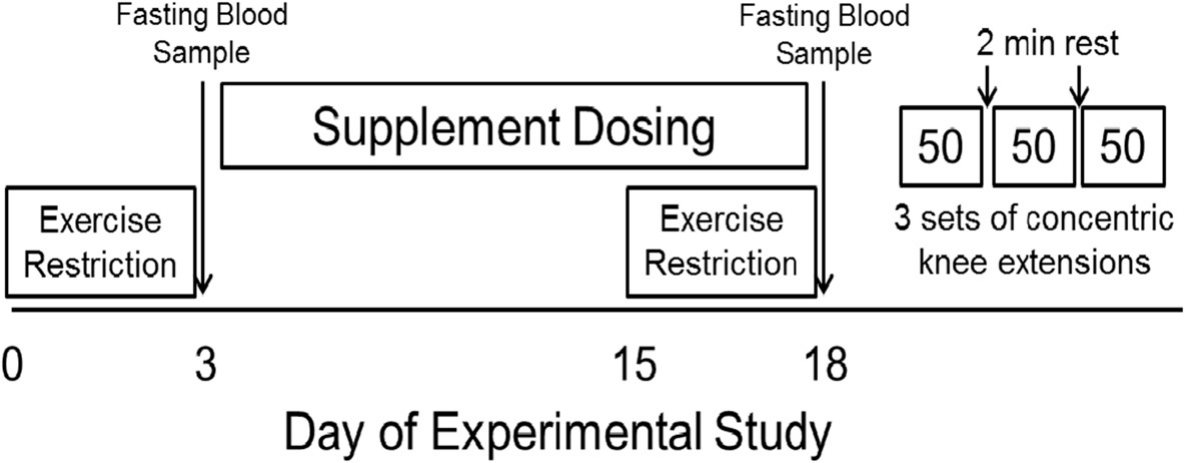
**Timeline of experimental procedures.** Each participant participated in two experimental trials, one for each treatment, separated by at least one week for supplement wash out and recovery. During each trial participants were assigned to either: (**a**) 15 days oral ingestion of placebo; or (**b**) 15 days oral ingestion of 400 mg ATP/d with the dosage divided into two equal dosages, one in the morning and the other in the evening.

Sixteen volunteers (8 male and 8 female; ages: 21–34 years) were enrolled in a double-blinded, placebo-controlled study using a crossover design. The protocol followed during each supplementation and testing period is shown in Figure 1. Both the placebo capsules containing rice flour and the ATP capsules containing 200 mg of Peak ATP® were obtained from a commercial manufacturer (TSI (USA), Inc., Missoula, MT). The ATP supplement was delivered as the disodium salt. A daily dosage of 400 mg/d was utilized for the current study and was chosen because the 225 mg ATP/d dosage used by Jordan et al. failed to improve bench press strength compared with the placebo group [21], and we reasoned that a higher dosage may be necessary to demonstrate an effect of oral ATP on knee extension fatigue and strength. A washout period of at least 1 week separated the experimental trials. For each of the trials, participants consumed their assigned capsules for 15 days as previously described. After the supplementation period, the participants reported to the laboratory for testing after an overnight fast of 12 h. The participants were given a supply of capsules (each containing 200 mg per capsule) and instructed to take two capsules each day (total dose = 400 mg/day), one in the morning before breakfast and one in the evening before dinner. 

All of the participants were classified as healthy and were not currently taking prescription medications or other dietary supplements. Multi-vitamins not exceeding the RDA were allowed. None of the participants were classified as competitive athletes or currently participated in daily heavy physical work or weight training. Participants had to be able to perform the fatigue testing and also were required to commit to maintaining their current activity levels throughout the study. Participants also had to agree to repeat a consistent dietary intake for the 24-hour period before each of the testing protocols. Participants not able to meet the inclusion criteria were excluded from the study. All procedures involving human participants were approved by the Iowa State University Institutional Review Board, and written informed consent was obtained from all participants prior to participation.

For each of the trials, participants refrained from vigorous exercise for three days before reporting to the laboratory in the morning after an overnight fast (Figure 1). Exercise consisting of light stretching and/or mild aerobic exercise lasting less than 45 minutes was allowed during this pre-study period. At this time, a blood sample was obtained. Weight and height were measured and BMI was calculated. Additionally, for characteristic purposes only, body composition was measured using air displacement plethysmography (BodPod®, Life Measurements, Concord, CA). The participants were then given their first week supply of blinded capsules with instructions on proper dose scheduling and completion of a dose-log. Participants returned to the laboratory after the first week to receive their second week of capsules and to confirm their compliance with the dosing schedule; there were no training or nutrition journals recorded.

At the end of the 15 days of dosing, the participants returned to the laboratory for post-supplementation testing. Another blood sample was taken and the participant’s body weight was again measured and BMI calculated. The participants were allowed to recover from the blood sampling for at least 30 min and then the strength/fatigue testing measurements were taken. No supplement was given before testing and all testing was conducted after an overnight fast and after three days of exercise restriction as in the preliminary testing. At the completion of each supplementation period, the strength/fatigue testing was performed which consisted of three 50-contraction knee extension fatigue tests conducted on a Biodex® leg dynamometer (BIODEX® Multi-Joint Systems 2: Biodex Medical version 4.5 software) with 2 minutes of rest between the tests. During each of the fatigue tests, the participants were instructed to extend the knee with maximum effort at a speed of 120 degrees per second. Peak torque of each individual contraction was recorded. The high peak torque is the maximum force generated during each of the 50 contractions, while the low peak torque would be the value of the lowest peak torque produced during the last ten contractions of each of the 50 contraction fatigue test. Work performed and average power in the 50 contractions were also measured. Percent fatigue was calculated as the percentage decline in high peak to low peak torque, and percent work fatigue was calculated as the percentage decrease in the work performed from the 1^st^ one-third to the last one-third of the contractions of each set. After a one week wash out and recovery period, the participants were switched to the other treatment and the testing was repeated (Figure 1). Blood samples were taken from a superficial forearm vein before and after each supplementation period and sent to a commercial laboratory for blood chemistry and complete blood count (LabCorp, Kansas City, MO).

### Statistics

A cross-over, repeated measures ANOVA model was used to analyze the data using the General Linear Models (GLM) procedure in SAS (SAS Institute, Cary, NC). No priori power analysis was performed, but the number of participants studied was justified based upon the Jordan et al. study [21] and the present study used a similar number of each gender in our crossover design as no prior art specific to exogenous ATP provided evidence to warrant against the use of both men and women. Participants were randomly assigned to treatment order. Main effects of participant, order, treatment (Trt), and Trt*time were included in the model. Least Squares Means procedure was then used to compare treatment means of each set. Statistical significance was determined at p < 0.05 and trends were determined for p > 0.05 and p < 0.10.

## Results

Participant characteristics are shown in Table 1. There were no significant changes in participant characteristics over the two treatment periods.

**Table 1:** **Participant characteristics at baseline for Placebo and 400 mg ATP**/**d**.*

	**Placebo**	**400 mg ATP**/**d**
Body Weight, kg		
All	71.0±10.3	70.9±10.4
Females	67.3±10.8	67.4±10.4
Males	74.7±8.9	74.4±9.7
Body Fat, %		
All	18.9±8.3	18.7±9.8
Females	25.0±3.4	26.2±3.6
Males	12.9±7.2	11.2±8.0
Body Mass Index		
All	23.3±2.5	23.3±2.7
Females	23.3±2.9	23.3±3.0
Males	23.3±2.3	23.2±2.5

^*^Studies were carried out on 16 participants (8 males and 8 females) with a mean age of 25.3 ± 3.9 years. Data are expressed as mean ± SD.

**Figure 2:**
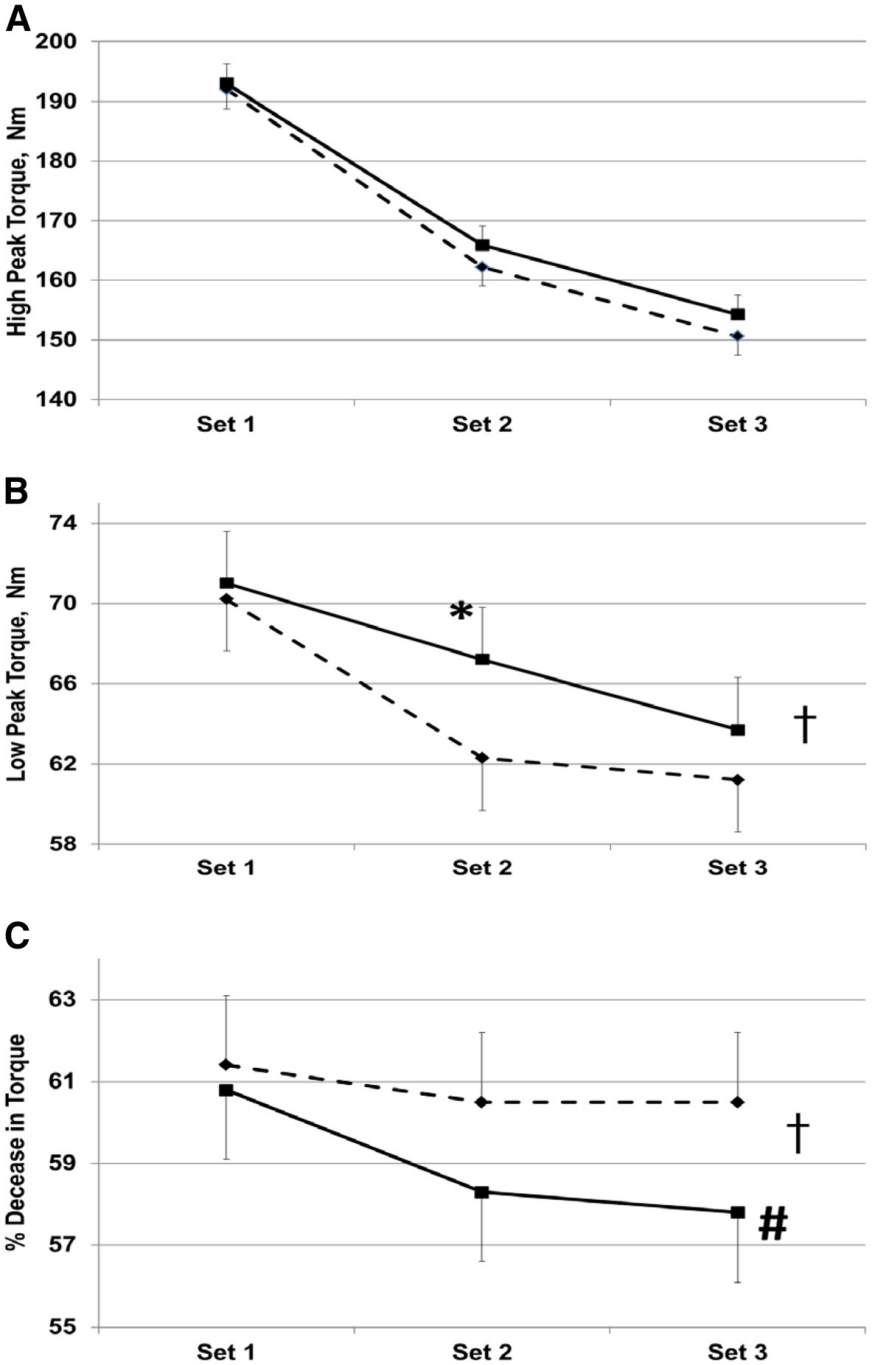
**High Peak Torque** (**A**); **Low Peak Torque** (**B**) **and Torque Fatigue** (**C**) **over 3 successive sets of 50**-**contraction knee extensions in Placebo** - - ♦- - **and 400 mg ATP**/**d** —▪— **supplemented participants.** Treatment with ATP approached an overall treatment main effect over placebo supplementation for Low Peak Torque and Torque Fatigue (**B** and **C**, † p < 0.11). ATP supplementation resulted in a significant improvement in Set 2 Low Peak Torque (**B**, * p < 0.01) and a trend for less Torque Fatigue in Set 3 (**C**, # p < 0.10).

High peak torque, low peak torque, and torque fatigue of the leg muscles measured over the three exercise sets are shown in Figure 2. No significant difference was found for high peak torque (Figure 2A). Overall treatment main effect of supplementing 400 mg of ATP approached significance for both increased low peak torque (Figure 2B) and decreased torque fatigue (Figure 2C). Analysis (Least Squares Means) of the data by each set showed that ATP supplementation significantly increased low peak torque in set 2 (62.3 and 67.2 Nm in placebo- and ATP-supplemented participants, respectively (p < 0.01)). Set 3 torque fatigue also tended to be less with ATP-supplementation (60.5% and 57.8% in placebo- and ATP-supplemented participants, respectively (p < 0.10)). However, the improvements seen in leg low peak torque did not lead to increased leg average power, total work, or a decrease in work fatigue.

Blood chemistries and differential cell counts were measured before and after each supplementation period. While some measurement comparisons between placebo and ATP-supplemented participants showed numerical differences that were statistically significant, none of the significant observations were clinically relevant and these data showed no untoward effects of the supplementation (data shown in Additional file 1: Table S1 and Table S2).

## Discussion

The current study shows that 400 mg ATP per day was effective in improving leg muscle low peak torque in set 2 (p < 0.01), and tended to decrease leg muscle fatigue in set 3 (p < 0.10) of three successive sets of knee extension exercises. However, the improvement in low peak torque and decreased fatigue were not sufficient to translate into improvements in leg muscle power or work performed. These observations lead us to speculate that supplemental ATP may provide cumulative benefits in strenuous, repetitive, and exhaustive exercise activities, which could lead to improved strength and lean body mass gains.

There is limited human data related to the potential for oral ATP to manifest physiologic modifications that would improve skeletal muscle efficiency or work performed [21]. As muscle undergoes prolonged work, ATP synthesis increases in an attempt to keep up with energy demand [22]. To accomplish this, the muscle needs substrates, such as oxygen and glucose, supplied from the peripheral circulation. Endogenous muscle stores of ATP are limited and support maximal work for only a fraction of, or at most 1–2 seconds and is replenished by the supply of intercellular phosphocreatine for only an additional 2–7 seconds [7]. Muscle performing exhaustive exercise then relies primarily on anaerobic glycolysis for regeneration of ATP which results in the production of lactate and H^+^. The associated decrease in intracellular pH is a factor leading to muscle fatigue [23,24]. Therefore, during maximal exertion blood flow is needed not only for oxygen supply to support continued oxidative phosphorylation, but also for H^+^ removal for muscle pH regulation. It would seem that exogenous ATP would likely have a greater impact on the muscles’ ability to perform fatiguing exercise by increasing substrate availability to the muscle and/or facilitating waste product removal through increased blood flow through the muscle tissues.

Both ATP and adenosine can act through purinergic receptors in endothelial smooth muscle of the vascular system resulting in vasodilation and increased blood flow [14,15,25]. A study by Gonzalez-Alonso showed that intra-arterial infusion of ATP was associated with vasodilation and increased blood flow with a significant reduction in venous ATP levels in the non-exercising limb suggesting utilization of ATP or metabolites [26]. These observations were confirmed by Calbet et al. who hypothesized that increased delivery of ATP would affect non-exercising vasoconstrictive muscle tissue [20]. These are most likely due to activation of purinergic receptors affecting blood flow [13]. Furthermore, exogenous adenosine administration results in vasodilation [14] and increased glucose and O_2_ uptake by muscle which provide for an increased substrate pool [12]. The ATP used in the present study was not enterically coated and was fed encapsulated as the disodium salt. The sodium salt would have provided buffering of the ATP through the stomach and the ATP itself should have been metabolically available as soon as it reached the proximal duodenum, which has been shown to be the most biologically active site for ATP metabolism and/or absorption [17]. In France, this chemical form of ATP is approved as a drug for lower back pain [27,28]. One proposed mechanism of action is through improved oxygenation of the muscle, which could be of similar benefit during exhaustive exercise.

Other effects of ATP or its metabolites could also indirectly impact work performance as ATP has immunomodulatory effects [29], and inotropic effects on cardiac muscle [30,31]. Oral administration of ATP to rabbits for 14 days results in systemic effects through a reduction in peripheral vascular resistance, improvement of cardiac output, reduction of lung resistance, and increased arterial PaO_2_[32]. A study in humans demonstrated that interstitial infusion of adenosine in muscle induced nitric oxide formation in skeletal muscle and nitric oxide and prostacyclin formation in microvascular endothelial cells [15]. Alternatively, the effects of cbvexogenously administered ATP may also be due to the associated increase in plasma uric acid, which has been proposed to act as an anti-oxidant [33,34]. Increased plasma uric acid has been demonstrated with ATP supplementation [17,18]. These studies may indicate further metabolism of adenosine before becoming bioavailable and warrant further investigation. These effects of ATP and adenosine could account, at least in part, for the improvements in low peak torque and torque fatigue we observed. The current study tested the hypothesis that oral ATP could improve performance during high intensity exercise. While we have shown this may be possible, the current study did not utilize methodologies to investigate the potential mechanism for the effects we observed. Further studies should incorporate measures of ATP and metabolites in blood components, should include measures of blood oxygenation and muscle blood flow, and also should investigate the extracellular role of ATP on the neuromuscular junction via Ca^2+^ mediated effects [35] as indicators of the potential mechanism by which oral ATP affects the ability to perform strenuous exercise.

Our study, like others in the literature, has limitations. The number of participants in the present study (n=16), while higher than that (n=9) previously studied by Jordan et al. [21], may not be sufficient to answer all the questions needed to validate the findings. Another limitation may relate to the timing of the last dose of oral ATP (or placebo) given. In our study the last dose was consumed 12 hours prior to testing. This contrasts with the study by Jordan et al. who studied participants after 14 days of supplementation and 3 hours post supplement dosing, and found ATP increased within group set 1 repetitions and total lifting volume [21]. Another potential limitation is that the study involved eumenorrheic females who were not differentiated based upon phase of the menstrual cycle. Other potential limitations include participants’ potential variation in physical activity or diet before testing. However, participants did commit to maintaining their physical activity level for the duration of the study and to exercise restrictions for 3 days prior to testing which within a crossover design should have minimized the effect of activity on the results. Additionally, participants were required to repeat a similar dietary intake 24 h before each testing period and the testing was performed after an overnight fast which should have minimized any acute dietary effect on testing results.

## Conclusions

In conclusion, the current study demonstrated that supplementation with 400 mg ATP/d for 15 days tended to reduce muscle fatigue while improving muscle low peak torque through successive sets of exhaustive exercise. These effects may indicate an improvement in overall training stimulus which may have been brought about by more rapid repolarization and stronger action potentials later within sets, which should be investigated further.

## Abbreviations

ATP: Adenosine-5’-triphosphate; BMI: Body Mass Index.

## Competing interests

This research was funded in part through a grant from the Grow Iowa Values Fund to Metabolic Technologies, Inc., Ames, IA, and in part by TSI (USA), Inc., Missoula, MT. The study was listed at ClinicalTrials.gov (NCT01141504). TSI (USA), Inc. also provided the Peak ATP® and placebo supplements used in the study. RS and HA declare no competing interests. JR, JF, and SB are employed by Metabolic Technologies, Inc which engages in business trade with TSI (USA), Inc. NA is a part owner of Metabolic Technologies, Inc.

## Authors’ contributions

RS was the principle investigator of the study and designed the study. RS and HA implemented the study and collected the data. JR, SB, NA, and RS participated in the design of the study and in the writing of this manuscript. JR and JF performed data analysis and JF wrote the manuscript. All authors read and approved the final manuscript.

## Supplementary Material

Additional file 1 **Table S1.** Blood chemistry values before and after 15 days of supplementation with either a placebo or 400 mg ATP/d.^*^Table S2. Blood hematology values before and after 15 days of supplementation with either a placebo or 400 mg ATP/d.^* ^ Click here for file
