# Supplementary material for: Adenosine-5'-triphosphate (ATP) supplementation improves low peak muscle torque and torque fatigue during repeated high intensity exercise sets
Source: J Int Soc Sports Nutr. 2012 Oct 9;9:48. doi: 10.1186/1550-2783-9-48 (PMC3483284; doi:10.1186/1550-2783-9-48)
Supplement: Additional file 1 — Table S1. Blood chemistry values before and after 15 days of supplementation with either a placebo or 400 mg ATP/d.*Table S2. Blood hematology values before and after 15 days of supplementation with either a placebo or 400 mg ATP/d.* [file 1550-2783-9-48-S1.docx]

**Supplemental Table 1.** Blood chemistry values before and after 15 days of supplementation with either a placebo or 400 mg ATP/d.^*^

|  | Placebo | | | | |  | 400 mg ATP | | | | |
| --- | --- | --- | --- | --- | --- | --- | --- | --- | --- | --- | --- |
|  | Before | SE^†^ | After | SE | % Change |  | Before | SE | After | SE | % Change |
| Glucose, mmol/l | 4.85 | 0.10 | 4.60 | 0.07 | -5.2 |  | 4.49 | 0.24 | 4.78 | 0.05 | 6.5 |
| Uric Acid, mmol/l^‡^ | 0.30 | 0.01 | 0.28 | 0.02 | -5.0 |  | 0.24 | 0.04 | 0.30 | 0.04 | 21.3 |
| Blood Urea Nitrogen, mmol/l | 5.29 | 0.32 | 5.22 | 0.34 | -1.3 |  | 5.31 | 0.30 | 4.64 | 0.22 | -12.6 |
| Creatinine, μmol/l | 76.6 | 3.1 | 76.4 | 2.9 | -0.4 |  | 75.1 | 3.0 | 75.8 | 3.0 | 0.9 |
| Sodium, mmol/l | 141.3 | 0.7 | 141.3 | 0.5 | 0.0 |  | 141.2 | 0.6 | 141.4 | 0.7 | 0.2 |
| Potassium, mmol/l | 4.57 | 0.11 | 4.63 | 0.14 | 1.4 |  | 4.59 | 0.14 | 4.68 | 0.15 | 1.8 |
| Chloride, mmol/l | 104.0 | 0.5 | 103.3 | 0.6 | -0.7 |  | 103.4 | 0.6 | 103.7 | 0.6 | 0.2 |
| CO_2_, mmol/l | 26.1 | 0.6 | 26.9 | 0.5 | 3.1 |  | 25.7 | 0.6 | 26.8 | 0.6 | 4.4 |
| Phosphorus, mmol/l^‡^ | 1.34 | 0.06 | 1.33 | 0.08 | -0.7 |  | 1.25 | 0.07 | 1.27 | 0.05 | 1.0 |
| Calcium, mmol/l | 2.36 | 0.03 | 2.38 | 0.03 | 0.9 |  | 2.36 | 0.03 | 2.36 | 0.03 | 0.1 |
| Protein, g/l | 68.1 | 1.6 | 69.1 | 1.6 | 1.5 |  | 67.5 | 1.6 | 68.1 | 1.5 | 0.9 |
| Albumin, g/l | 43.4 | 0.8 | 44.9 | 0.7 | 3.6 |  | 44.5 | 0.8 | 44.1 | 0.8 | -0.8**^#^** |
| Globulin, g/l | 24.7 | 1.1 | 24.1 | 1.1 | -2.3 |  | 23.0 | 1.0 | 24.0 | 0.9 | 4.4 |
| A:G Ratio | 1.8 | 0.1 | 1.9 | 0.1 | 6.9 |  | 2.0 | 0.1 | 1.9 | 0.1 | -5.6**^#^** |
| Total Bilirubin, μmol/l | 8.6 | 1.0 | 9.6 | 0.9 | 12.5 |  | 9.9 | 1.0 | 8.7 | 1.1 | -12.9 |
| Direct Bilirubin, μmol/l^‡^ | 1.90 | 0.14 | 2.55 | 0.13 | 34.2 |  | 2.55 | 0.31 | 1.85 | 0.15 | -27.5**^#^** |
| Alkaline Phosphatase, IU/l | 55.8 | 2.7 | 57.9 | 3.4 | 3.9 |  | 57.1 | 3.1 | 58.4 | 3.8 | 2.2 |
| Lactate Dehydrogenase, IU/l | 153.9 | 4.2 | 170.5 | 19.5 | 10.8 |  | 153.1 | 6.7 | 169.0 | 18.4 | 10.4 |
| Aspartate Aminotransferase, IU/l | 19.9 | 1.4 | 20.3 | 1.0 | 1.9 |  | 20.0 | 0.9 | 22.4 | 2.5 | 11.9 |
| Alanine Aminotransferase, IU/l | 18.9 | 2.2 | 17.2 | 1.5 | -9.2 |  | 16.6 | 1.2 | 18.1 | 1.7 | 9.4**^#^** |
| Gamma-glutamyl  Transpeptidase, IU/l^‡^ | 16.8 | 2.1 | 16.9 | 2.4 | 0.6 |  | 19.2 | 2.2 | 19.5 | 2.8 | 1.6 |
| Iron Binding Capacity, μmol/l^‡^ | 60.9 | 2.8 | 64.4 | 3.2 | 5.8 |  | 60.3 | 2.9 | 60.3 | 2.8 | 0.2 |
| UIBC, μmol/l^‡^ | 47.7 | 3.4 | 44.7 | 4.4 | -6.4 |  | 41.2 | 4.4 | 47.6 | 2.9 | 15.4 |
| Iron, μmol/l^‡^ | 13.2 | 1.7 | 19.7 | 2.3 | 49.9 |  | 19.0 | 2.3 | 12.8 | 1.6 | -32.8**^#^** |
| Iron Saturation, %^‡^ | 22.1 | 2.9 | 31.7 | 3.9 | 43.4 |  | 33.0 | 4.8 | 21.3 | 2.6 | -35.5**^#^** |
| Total Cholesterol, mmol/l | 4.34 | 0.12 | 4.54 | 0.13 | 4.7 |  | 4.30 | 0.09 | 4.31 | 0.09 | 0.3 |
| Triglycerides, mmol/l | 1.16 | 0.12 | 1.22 | 0.11 | 5.1 |  | 1.12 | 0.12 | 1.15 | 0.08 | 2.1 |
| HDL, mmol/l | 1.38 | 0.07 | 1.39 | 0.06 | 0.1 |  | 1.40 | 0.07 | 1.44 | 0.06 | 2.5 |
| LDL, mmol/l | 2.42 | 0.13 | 2.60 | 0.15 | 7.3 |  | 2.38 | 0.11 | 2.37 | 0.09 | -0.7 |
| Cholesterol Ratio | 3.21 | 0.14 | 3.34 | 0.15 | 4.1 |  | 3.18 | 0.17 | 3.06 | 0.11 | -3.9 |

^*^Fasting blood samples were taken before and after the 15 day supplement period. Results are the means of 16 subjects, 8 males and 8 females except where indicated.

^†^Standard error of the mean.

^‡^Measured in first 10 subjects only.

^#^Significant difference for % change in the placebo versus % change in the ATP group p < 0.05.

**Supplemental Table 2.** Blood hematology values before and after 15 days of supplementation with either a

placebo or 400 mg ATP/d.^*^

|  | Placebo | | | | |  | 400 mg ATP | | | | |
| --- | --- | --- | --- | --- | --- | --- | --- | --- | --- | --- | --- |
|  | Before | SE^†^ | After | SE | % Change |  | Before | SE | After | SE | % Change |
| WBC, x10^9^/l | 7.0 | 0.3 | 7.1 | 0.2 | 1.9 |  | 6.3 | 0.2 | 6.6 | 0.2 | 5.6 |
| RBC, x10^12^/l | 4.5 | 0.1 | 4.6 | 0.1 | 1.6 |  | 4.5 | 0.1 | 4.5 | 0.1 | 0.7 |
| Hemoglobin, g/l | 140 | 2.5 | 142 | 2.6 | 2.0 |  | 140 | 2.7 | 140 | 2.1 | 0.5 |
| Hematocrit, l/l | 0.42 | 0.01 | 0.42 | 0.01 | 0.3 |  | 0.41 | 0.01 | 0.41 | 0.01 | -0.5 |
| MCV, μm^3^ | 92.6 | 0.9 | 91.4 | 0.8 | -1.2 |  | 92.4 | 0.9 | 91.2 | 0.8 | -1.3 |
| MCH, pg | 31.0 | 0.3 | 31.1 | 0.2 | 0.4 |  | 31.2 | 0.3 | 31.1 | 0.3 | -0.1 |
| MCHC, g/l | 335 | 1.4 | 341 | 1.2 | 1.8 |  | 338 | 1.4 | 341 | 1.1 | 1.0 |
| RDW, % | 13.7 | 0.2 | 13.7 | 0.1 | -0.1 |  | 13.6 | 0.2 | 13.5 | 0.1 | -0.5 |
| Platelets, x10^9^/l | 232 | 10.8 | 238 | 9.4 | 2.9 |  | 221 | 9.5 | 237 | 13.4 | 7.5 |
| Neutrophils, % | 52.4 | 1.4 | 49.1 | 1.6 | -6.3 |  | 46.4 | 2.0 | 47.6 | 1.8 | 2.6**#** |
| Lymphocytes, % | 36.5 | 1.7 | 40.2 | 1.5 | 10.2 |  | 42.2 | 1.9 | 40.8 | 1.7 | -3.4**#** |
| Monocytes, % | 8.0 | 0.4 | 7.9 | 0.5 | -2.1 |  | 8.2 | 0.4 | 8.7 | 0.6 | 5.4**#** |
| Eosinophils, % | 2.5 | 0.3 | 2.5 | 0.3 | -1.5 |  | 2.7 | 0.4 | 2.6 | 0.3 | -3.7 |
| Basophils, % | 0.6 | 0.1 | 0.4 | 0.1 | -31.8 |  | 0.5 | 0.1 | 0.5 | 0.1 | -9.3 |
| Neutrophils, x10^9^/l | 3.7 | 0.2 | 3.5 | 0.2 | -4.9 |  | 2.9 | 0.2 | 3.2 | 0.1 | 8.1**#** |
| Lymphocytes, x10^9^/l | 2.5 | 0.1 | 2.8 | 0.1 | 12.7 |  | 2.6 | 0.1 | 2.7 | 0.1 | 2.7 |
| Monocytes, x10^9^/l | 0.6 | 0.04 | 0.6 | 0.04 | -0.5 |  | 0.5 | 0.04 | 0.6 | 0.04 | 8.5**#** |
| Eosinophils, x10^9^/l | 0.2 | 0.02 | 0.2 | 0.02 | -4.4 |  | 0.2 | 0.02 | 0.2 | 0.02 | -10.7 |
| Basophils, x10^9^/l | 0.05 | 0.01 | 0.03 | 0.01 | -33.4 |  | 0.04 | 0.01 | 0.04 | 0.01 | -7.9 |

^*^Fasting blood samples were taken before and after the 15 day supplement period. Results are the means of 16

subjects, 8 males and 8 females.

^†^Standard error of the mean.

^#^Significant difference for % change in the placebo versus % change in the ATP group p < 0.05.
